# P-1102. Real-World Effectiveness of Dalbavancin for Invasive Gram-Positive Bacterial Infections

**DOI:** 10.1093/ofid/ofae631.1290

**Published:** 2025-01-29

**Authors:** Jake Crocker, Caroline Derrick, Julie Ann Justo, P Brandon Bookstaver, Ismaeel Yunusa, Bruce M Jones, Bryan White, Joseph Sassine, Madeline Belk, Morgan Pizzuti

**Affiliations:** Prisma Health Upstate, Columbia, SC; University of South Carolina School of Medicine, Columbia, South Carolina; Dartmouth Hitchcock Medical Center, East Thetford, Vermont; Prisma Health Richland - University of South Carolina, Columbia, South Carolina; University of South Carolina College of Pharmacy, Columbia, South Carolina; St. Joseph's/Candler Health System, Savannah, GA; University of Oklahoma Medical Center, Oklahoma City, Oklahoma; University of Oklahoma Health Sciences Center, Oklahoma City, Oklahoma; Huntsville Hospital, Huntsville, Alabama; Prisma Health Richland - University of South Carolina, Columbia, South Carolina

## Abstract

**Background:**

Emerging data suggest dalbavancin (DAL) may be used in complicated infections including bacteremia, prosthetic joints, osteomyelitis, and endovascular infections. The purpose of this study was to evaluate the real-world effectiveness of DAL for the treatment of invasive gram-positive infections (GPIs).

**Methods:**

This multicenter, retrospective cohort study included adult patients receiving DAL for treatment of confirmed/presumed invasive GPI between February 2021 and December 2023. Invasive infections included those requiring ≥ 4 weeks of therapy. Patients were excluded if they received DAL for chronic suppression. The intention to treat (ITT) population included patients who received ≥ 1 DAL dose; the per protocol (PP) population received ≥ 2 doses within 7 days of expected administration dates. The primary outcome was clinical failure in the PP group at 90 days post-treatment. Secondary outcomes were logistical failures, clinical failures, and treatment-emergent adverse events in the ITT group at 90 days post-treatment.

**Results:**

The ITT population included 150 patients (mean age 50.2 years, 65% male, 35% obese, 41% illicit drug use); the PP population included 107 patients. The most common infectious syndrome was osteomyelitis (63.3%, 95/150); the most common pathogens were MRSA (38%, 57/150) and MSSA (37%, 44/150). The most common dosing regimen was 1500 mg on day 1, followed by 1500 mg on day 8 (90%, 96/150). Among the PP population with follow-up, 22% (19/87) experienced clinical failure at 90 days post-treatment. No factors were significantly associated with clinical failure. Logistical failure occurred in 17% (26/150). Results of univariate regression analyses showed receipt of first dose at an infusion center vs. inpatient setting significantly increased logistical failure (OR 4.53, 95% CI 1.62 – 16.17; *P* = 0.008) while blood-based infectious syndromes significantly decreased logistical failure (OR 0.26, 95% CI 0.06 – 0.82; *P* = 0.038). Receipt of vancomycin within 6 weeks of DAL significantly increased risk of acute kidney injury (OR 4.12, 95% CI 1.20 – 14.68; *P* = 0.024).

**Conclusion:**

Dalbavancin is a safe and effective treatment option for invasive GPIs; however there are modifiable risk factors associated with secondary outcomes of logistical failure and toxicity.

**Disclosures:**

**Julie Ann Justo, PharmD, MS, FIDSA, BCPS**, Shionogi: Advisor/Consultant **Bruce M. Jones, Pharm.D., FIDSA, BCPS**, AbbVie: Advisor/Consultant|AbbVie: Honoraria|Ferring: Honoraria|Innoviva: Honoraria|Paratek: Honoraria **Bryan White, PharmD, BCPS, BCIDP**, Melinta: Advisor/Consultant|Shionogi: Grant/Research Support **Joseph Sassine, MD**, Ansun BioPharma: Grant/Research Support|Cidara Therapeutics: Grant/Research Support|Community Infusion Solutions: Grant/Research Support|F2G: Grant/Research Support|Shionogi: Grant/Research Support

